# Objective Knowledge Mediates the Relationship between the Use of Social Media and COVID-19-Related False Memories

**DOI:** 10.3390/brainsci11111489

**Published:** 2021-11-11

**Authors:** Chiara Scuotto, Ciro Rosario Ilardi, Francesco Avallone, Gianpaolo Maggi, Alfonso Ilardi, Giovanni Borrelli, Nadia Gamboz, Marco La Marra, Raffaella Perrella

**Affiliations:** 1 Department of Psychology, University of Campania Luigi Vanvitelli, 81100 Caserta, Italy; chiara.scuotto1@gmail.com (C.S.); cirorosario.ilardi@unicampania.it (C.R.I.); gianpaolo.maggi@unicampania.it (G.M.); giovanni.borrelli.1991@virgilio.it (G.B.); raffaella.perrella@unicampania.it (R.P.); 2 Department of Family Medicine, McGill University, Montreal, QC H3S1Z1, Canada; francesco.avallone@mail.mcgill.ca; 3 Inmates Ward, Department of Internal Medicine, Antonio Cardarelli Hospital, 80131 Naples, Italy; alfonso.ilardi@aocardarelli.it; 4 Laboratory of Experimental Psychology, Suor Orsola Benincasa University, 80135 Naples, Italy; nadia.gamboz@unisob.na.it; 5 Department of Experimental Medicine, University of Campania Luigi Vanvitelli, 80138 Naples, Italy

**Keywords:** false memories, COVID-19, fake news, fear, objective knowledge, social media

## Abstract

The exposure to relevant social and/or historical events can increase the generation of false memories (FMs). The Coronavirus Disease 2019 (COVID-19) pandemic is a calamity challenging health, political, and journalistic bodies, with media generating confusion that has facilitated the spread of fake news. In this respect, our study aims at investigating the relationships between memories (true memories, TMs vs. FMs) for COVID-19-related news and different individual variables (i.e., use of traditional and social media, COVID-19 perceived and objective knowledge, fear of the disease, depression and anxiety symptoms, reasoning skills, and coping mechanisms). One hundred and seventy-one university students (131 females) were surveyed. Overall, our results suggested that depression and anxiety symptoms, reasoning skills, and coping mechanisms did not affect the formation of FMs. Conversely, the fear of loved ones contracting the infection was found to be negatively associated with FMs. This finding might be due to an empathy/prosociality-based positive bias boosting memory abilities, also explained by the young age of participants. Furthermore, objective knowledge (i) predicted an increase in TMs and decrease in FMs and (ii) significantly mediated the relationships between the use of social media and development of both TMs and FMs. In particular, higher levels of objective knowledge strengthened the formation of TMs and decreased the development of FMs following use of social media. These results may lead to reconsidering the idea of social media as the main source of fake news. This claim is further supported by either the lack of substantial differences between the use of traditional and social media among participants reporting FMs or the positive association between use of social media and levels of objective knowledge. The knowledge about the topic rather than the type of source would make a difference in the process of memory formation.

## 1. Introduction

The fabricated or distorted recollection of an event is defined as a false memory (FM). People may remember events that never happened or may remember them differently from how they actually occurred [1]. Intriguingly, the rate of FMs seems to be affected by specific individual variables, e.g., knowledge (alleged or actual) about the topic of interest, emotional arousal and valence, depressive and anxiety symptoms, personality factors, and cognitive abilities.

FMs appear to be related to a higher interest in, and engagement with, a certain topic [2,3], which underlines a greater exposure to news on the matter. In this respect, as recently observed, FMs were found to be associated with the spreading of fake news [4]. Some studies found that FMs are experienced more frequently by individuals exaggerating their perceived knowledge about the topic of interest (i.e., overclaiming). This finding is likely explained by the tendency to not admit that they do not remember a given piece of information due to social pressure [5]. Objective knowledge about a certain topic has instead been associated with a lower frequency of FMs [6,7]. Indeed, people would tend to form high-level cognitive reasoning schemes for topics on which they have high knowledge about; such a tendency may represent a protective factor against the development of memory biases [8]. This hypothesis is in accordance with the rationale of the emerging Monitoring Model Framework (MMF) [9,10]. According to the MMF, people would discriminate a true memory (TM) from a FM by evaluating their personal mental experiences based on two cognitive systems operating in parallel. The first system is based on heuristic judgments employed to determine whether the contents of an alleged memory correspond to the typical features (e.g., perceptual, temporal, and semantic) of a TM. For instance, TMs are usually more vivid and familiar than FMs, and they contain more spatial and temporal details compared with imagined or unreal experiences. Conversely, the second system is based on a more systematic approach according to which the contents of the alleged memory are carefully evaluated to verify their plausibility by comparing them with pre-existing knowledge and/or other (dis)confirmative evidence [10].

Emotions can also affect susceptibility to FMs. For instance, in the context of emotional arousal, some studies showed that individuals generally pay more attention to emotional than neutral events. As a consequence, emotional events are more likely to be encoded in memory. Once encoded, stress hormones in the brain consolidate emotional memories by making them more enduring [11,12]. This psychophysiological mechanism appears to significantly reduce people’s susceptibility to FMs [13]. However, it is important to underline that also emotional valence may play a role in the genesis of FMs. Indeed, it has been suggested that negative emotions lead to careful monitoring of the environment for encoding and processing relevant information to cope with unexpected scenarios. Conversely, people experiencing positive emotions seem to match to a less detail-oriented information-processing style, pending more relevant and/or rewarding stimuli within a benign and predictable environment. This results in the activation of re-constructive processes depending on existing knowledge and schematic heuristics [14]. In sum, people experiencing positive emotions may be more vulnerable to incorporate misleading information into memory than people experiencing negative emotions [12]. 

Studies exploring the relationship between FMs and levels of depression and anxiety provided mixed results. Some evidence suggested a negative association between depression and FMs and a positive association between anxiety and FMs [15,16]. Other investigations showed instead a positive relationship between depression and FMs formation [17]. 

Coping mechanisms, i.e., personality traits modulating the individual’s behavior in response to stressors, may also play a role in the development of FMs, although the available data are inconsistent. For instance, avoidance-coping strategies including massive stressor avoidance, in combination with denial and wishful thinking, were found to predict FMs construction [18]. Subsequent evidence has instead observed that negative coping strategies, such as avoidance and denial, were negatively associated with FMs; conversely, active coping strategies (e.g., positive reframing, planning) showed a positive association with the frequency of FMs [16]. More recently, some studies have reported that excessive use of help-seeking strategies (e.g., asking for advice, delegating to solve a problem, seeking social and moral support), in synergy with high-stress levels, would tend to facilitate FM formation [19,20]. 

Finally, high analytic skills, e.g., logical reasoning and critical thinking, may allow people to effectively monitor the sources of their memories, thus reducing the frequency of FMs [21]. In line with this claim, poor cognitive functioning has been associated with a higher likelihood of developing FMs [16]. However, contradictory findings are also available [22,23,24]. 

As widely acknowledged, the exposure to relevant social or historical events may enhance the tendency of generating FMs [25,26,27]. Clearly, the Coronavirus Disease 2019 (COVID-19) pandemic is a major, unpredictable, and unprecedented calamity heavily challenging health, political, administrative, and journalistic authorities [28,29]. Although the psychological symptoms experienced by individuals during the quarantine have led to the development of severe attentional and memories difficulties per se [30,31], the probability to develop FMs has been increased also by the resulting generalized confusion in media communication, a fertile ground for the spreading of misinformation and fake news [3,32]. Fake news, as antecedents, and FMs as consequences, can affect the creditability of the healthcare system and reduce the adherence to public health guidelines [33], such as those relating to the vaccination plan [34,35]. Therefore, especially in the current historic period, it would have been relevant to investigate the relationship between individual variables, including those described above, and susceptibility to FMs after exposure to COVID-19 fake news. To the best of our knowledge, only one study addressed this issue [7]. 

The aim of this study was to investigate, in a sample of Italian university students, whether FM development is affected by individual/personological variables in terms of use of traditional and social media, COVID-19 perceived and objective knowledge, fear of the disease, depression and anxiety symptoms, reasoning skills, and coping mechanisms. As unfiltered, rough, or false information is often spread through social media [36,37], it is reasonable to hypothesize that a higher use of social media facilitates the generation of FMs, particularly in young individuals [38]. However, higher objective knowledge about COVID-19 could represent a significant mediator across this relationship, with more knowledgeable individuals being less susceptible to FMs following the use of social media.

## 2. Materials and Methods

### 2.1. Participants

G*Power 3.1.9.4 was used to perform an a priori power analysis for determining the number of participants needed according to linear regression. At a nominal alpha level of 0.05, power (1 − β) set to 0.80, large effect size (*f*^2^ = 0.35), and a number of predictors ranging from 1 to 14, the required total sample size was between 25 and 66. 

Convenience sampling method was used. Participants were recruited via web through email, social media posts, and word-of-mouth. A total of 205 Italian University students were surveyed, 200 of whom completed the experimental protocol in full (97.6%, 150 females). Participants were attending the degree courses in a variety of disciplines including medicine, psychology, engineering, and economics. 

### 2.2. Procedure

Participants filled in a survey in the Italian language, which was constructed using the Google Forms platform. Respondents took about 25 min to complete the questionnaire. In the months of February and March 2021, the study rationale and methodological approach were defined. Data collection started on 16 April 2021 and ended on 8 May 2021. The survey consisted of several blocks. 

First, participants were informed about the study aim, i.e., investigating knowledge and memories about COVID-19 and their association with certain individual variables. Then, participants were asked to provide demographic information (i.e., sex, age, and years of education) and whether they got the Coronavirus infection. 

In the second block, participants were required to rate, on a 6-point Likert scale, the frequency of use of traditional media (e.g., newspaper articles, television) and social media (e.g., Facebook, Instagram, Twitter) for acquiring information about COVID-19 news and discussion (0 = “*not at all*”, 5 = “*a lot*”). Furthermore, they reported how afraid they were of contracting, and that their loved ones contracting, the virus (0 = “*not at all*”, 5 = “*a lot*”). Finally, perceived knowledge (PK) about COVID-19 was assessed by asking participants to judge, on the same 0–5 Likert scale, how competent they considered themselves to be about COVID-19 (in terms of studies, events, statistics, and political/administrative measures). 

In the third block, COVID-19 objective knowledge (OK) was measured through an ad hoc devised questionnaire consisting of eight statements (4 true and 4 false) about COVID-19 (true, e.g., “*The first clinical manifestations of the infection are generally fever, dry cough and muscle pain*”, “*Women are less affected by the infection compared with men*”; false, e.g., “*Quarantine is a precautionary isolation period lasting 40 days*”, “*There are many drugs that are effective in treating the infection*”). The questionnaire was constructed by a pneumologist in cooperation with two psychologists experienced in psychometrics and research methodology. One point was given for each statement correctly recognized as true or false (maximum OK score = 8). 

In the fourth block, levels of depression and anxiety were assessed by using the Beck Depression Inventory (BDI [39,40]) and the Beck Anxiety Inventory (BAI [41]), respectively. The BDI is a self-report questionnaire consisting of 21 statements exploring overt behavioral manifestations and symptomatic areas of depression in the last weeks. The severity of symptoms is evaluated on a Likert scale ranging from 0 to 3, with a higher score indicating higher levels of depression [41]. The BAI is a self-report questionnaire devised for the quantitative assessment of anxiety symptoms that occurred during the past week. The inventory consists of 21 items describing the emotional, physiological, and cognitive symptoms of anxiety. Each item is rated on a 0–3 Likert scale, with a higher score reflecting severer anxiety symptoms [42]. 

In the fifth block, coping mechanisms were measured through the COPE-New Italian Version (COPE-NVI-25 [43,44,45]). This is a 25-item tool assessing the frequency of use of coping mechanisms in difficult or stressful situations. Items are organized in five subscales: avoidance strategies (5 items), transcendent orientation (4 items), positive attitude (6 items), social support (5 items), and problem solving (5 items). The response format for each item is based on a Likert scale ranging from 1 (“*I do not usually do this*”) to 6 (“*I do it almost all the time*”). 

In the sixth block, participants completed the Italian version of the Cognitive Estimation Task (CET [46]). This task was devised to test reasoning and self-monitoring abilities. It is composed of 21 questions that are not immediately answerable; however, the response can be provided using previous knowledge. According to the absolute error scoring procedure, participants were awarded points, from 0 to 2, depending on the accuracy of the estimates provided: the higher the score, the poorer the performance. Bizarre responses are scored 0 or 1 (see Della Sala et al. [46] for additional details). 

In the last block, eight COVID-19-related news were presented. Four were fake news (e.g., *Flu vaccine* news, “*A recent study conducted by the Agostino Gemelli University Polyclinic in Rome has shown that 85% of patients who received flu vaccine were less likely to contract the COVID-19 for at least 4 months following the vaccination*”) and four were true news (e.g., *Surfaces* news, “*According to a recent Australian study published in the Virology Journal, a sample of SARS-CoV-2, in conditions of ideal humidity and temperature, survives on some surfaces such as glass, steel and banknotes for up to 28 days*”, see Appendix A). Fake news were totally fabricated and constructed after a careful examination of possible fake news spread on the web and television. Following this approach, the reporting of news items to which the subjects could have actually been exposed was prevented. Conversely, true news were extrapolated from information provided by the websites of the World Health Organization (WHO) and the Italian Ministry of Health. Participants were asked whether they remembered (“*I remember this*”) or not (“*I do not remember this*”) each news item. If participants reported remembering the news, they were asked to specify the source memory, i.e., where/how they acquired the information, i.e., traditional or social media. Note that each news was presented together with an image since previous studies showed that photographic stimuli tended to increase the generation of FMs [47]. One point was assigned for each remembered true/fake news, resulting in a True Memory (TM) and a False Memory (FM) score, which both ranged from 0 to 4. 

From the second to the sixth block, the order of presentation of blocks was randomized within the subjects. The eight news were also presented in random order. Informed consent was obtained from all the participants involved in the study immediately after the illustration of the study’s objectives. This study was performed according to the ethical standards as laid down in the 1964 Declaration of Helsinki and its later amendments and approved by the Ethics Committee of the University of Campania “Luigi Vanvitelli” (protocol code: 17/2021, date of approval by the Department of Psychology: 13 April 2021).

### 2.3. Statistical Analyses

First, we compared the response rates and the source type for each of the eight news item by means of one-way chi-squared test (χ^2^). The Phi coefficient (*φ* = $\sqrt{\chi^{2}/N}$) was used as a measure of the strength of the association between the two levels of each nominal variable and therefore to compute the χ^2^ effect size [48]. 

Then, Spearman’s correlation analysis (*r*_rho_) was used for exploratory purposes. More specifically, we correlated the TM and FM scores with the frequency of use of traditional and social media, fear of contracting—and fear that loved ones could contract—COVID-19, PK and OK scores, BDI and BAI scores, CET score, and the five COPE-NVI-25 sub-scores. The Cohen’s conventions [49] were used to interpret the effect size (weak, *r*_rho_ < 0.30; moderate, *r*_rho_ = 0.30–0.50; strong, *r*_rho_ > 0.50). 

Subsequently, we constructed fourteen simple linear regression models, where TM and FM scores entered individually into each model as dependent variables, whereas media usage, PK score, OK score, and all psychological variables were assumed as potential predictors. Hence, the variables that independently explained a sufficient proportion of variance of TM and FM scores were entered into a simultaneous multiple regression model in order to identify the most influential predictors of both TM and FM scores.

Finally, to investigate whether and how OK mediated the relationship between the frequencies of use of social and traditional media and the creation of TMs and FMs (i.e., TM and FM scores), we carried out two mediation analyses, entering TM score and FM scores as dependent variables, the frequencies of use of traditional and social media as predictors, and OK score as parallel mediator. In order to evaluate the significance of direct and indirect effects, bootstrapping procedure with 5000 samples with replacement from the full sample to construct bias-corrected 95% confidence intervals (hereafter 95% CI; LL = lower level of confidence interval, UL = upper level of confidence interval) was conducted by SPSS Macro PROCESS [50].

All statistical analyses were performed by IBM SPSS Statistics for Windows, version 26.0 (IBM, Armonk, NY, USA).

## 3. Results

### 3.1. Data Cleaning

Eight subjects were excluded due to a large number of missing values; moreover, 15 univariate outliers, i.e., subjects obtaining *z*-scores equal to, or greater than, 3 in absolute value were removed [51]. Descriptive statistics expressed as mean and standard deviation on a total of 177 participants (131 females; *M* age = 24.34, *SD* = 2.78, age range = 20–39; *M* years of schooling based on the degree awarded = 14.39, *SD* = 1.50, education range = 13–16) were computed for each variable under examination (see Table 1). Only 18 participants (8.8%) reported having caught the infection before participation in the study.

### 3.2. True and False Memories for COVID-19-Related News

Participants obtained a TM score significantly higher than the FM score (TM score, *M* = 1.89, *SD* = 0.97; FM score, *M* = 0.75, *SD* = 0.84; *t*_(176)_ = 12.181, *p* < 0.001). They also reported a more frequent use of traditional media rather than social media for news and insights regarding COVID-19 (traditional media, *M* = 3.56, *SD* = 1.19; social media, *M* = 3.18, *SD* = 1.37; *t*_(176)_ = 3.265, *p* = 0.001). 

The response rates for each true and fake news and information about source memory are summarized in Table 2. Overall, participants did not show a clear memory for the true news, except for the *Symptoms* news which was correctly recalled by 84.2% of participants (*n* = 177, χ^2^_(1)_ = 82.718, *p* < 0.001, *φ* = 0.68). As for the *Surfaces* news, about half of participants (53.7%) did not remember seeing/hearing it (*n* = 177, χ^2^_(1)_ = 0.955, *p* = 0.33); conversely, the majority of participants did not remember the *Immunization* (*n* = 177, χ^2^_(1)_ = 74.718, *p* < 0.001, *φ* = 0.65) and *Monoclonal antibodies* news (*n* = 177, χ^2^_(1)_ = 5.429, *p* = 0.02, *φ* = 0.17). Participants who correctly recalled the true news indicated having seen/heard them mainly through traditional media (*Immunization*, *n* = 31, χ^2^_(1)_ = 7.258, *p* = 0.007, *φ* = 0.48; *Monoclonal antibodies*, *n* = 73, χ^2^_(1)_ = 23.027, *p* < 0.001, *φ* = 0.56; *Symptoms*, *n* = 149, χ^2^_(1)_ = 23.362, *p* < 0.001, *φ* = 0.39), apart from the *Surfaces* news (*n* = 82, χ^2^_(1)_ = 1.756, *p* = 0.18). As concerns the fake news, most of the participants did not remember seeing/hearing them (*n* = 177, *Flu vaccine*, χ^2^_(1)_ = 50.989, *p* < 0.001, *φ* = 0.54; *Serological tests*, χ^2^_(1)_ = 67.124, *p* < 0.001, *φ* = 0.61; *Masks*, χ^2^_(1)_ = 135.734, *p* < 0.001, *φ* = 0.87; *Pets*, χ^2^_(1)_ = 40.819, *p* < 0.001, *φ* = 0.48). Participants who recalled the fake news, i.e., who reported FMs, indicated traditional media as primary source (69.3%) only for the *Flu vaccine* news (*n* = 41, χ^2^_(1)_ = 5.488, *p* = 0.02, *φ* = 0.36); for the remaining fake news, no difference was found between use of traditional and social media (*Serological tests*, *n* = 34, χ^2^_(1)_ = 1.059, *p* = 0.30; *Masks*, *n* = 11, χ^2^_(1)_ = 2.273, *p* = 0.13; *Pets*, *n* = 11, χ^2^_(1)_ = 0.348, *p* = 0.55). 

### 3.3. Correlation Analysis

TM score showed weak, but significant, positive correlations with use of social media (*r*_rho_ = 0.28, *p* < 0.001), fear of contracting COVID-19 (*r*_rho_ = 0.17, *p* = 0.02), and fear that loved ones contracting COVID-19 (*r*_rho_ = 0.18, *p* = 0.02). Furthermore, TM score correlated moderately, and positively, with use of traditional media (*r*_rho_ = 0.34, *p* < 0.001) and OK score (*r*_rho_ = 0.30, *p* < 0.001). FM score showed, instead, a negative moderate association only with OK score (*r*_rho_ = −0.35, *p* < 0.001). 

### 3.4. Predictors of True and False Memory Scores

As concerns the TM score, simple regression analysis revealed that higher use of traditional (*R^2^* = 0.13, *B* = 0.30, *p* < 0.001) and social media (*R^2^* = 0.08, *B* = 0.20, *p* < 0.001), higher OK score (*R^2^* = 0.08, *B* = 0.19, *p* < 0.001), greater fear of contracting (*R^2^* = 0.03, *B* = 0.16, *p* = 0.02) and loved ones contracting COVID-19 (*R^2^* = 0.03, *B* = 0.20, *p* = 0.03) predicted a significant increase in TM score. When these variables entered a simultaneous multiple regression analysis, only the use of traditional media (*B* = 0.24, *p* < 0.001), use of social media (*B* = 0.13, *p* = 0.009), and OK score (*B* = 0.13, *p* = 0.004) remained significant, with the regression model that was able to account for 24% of the variance in TM score (*F*_(5, 171)_ = 11.031, *p* < 0.001, see Table 3).

As concerns the FM score, simple regression analysis revealed that a higher OK score (*R^2^* = 0.15, *B* = −0.22, *p* < 0.001) and a greater fear that loved ones would contract COVID-19 (*R^2^* = 0.02, *B* = −0.16, *p* = 0.05) predicted a significant decrease in FM score. In multiple regression analysis, the OK score remained the only significant predictor (*B* = −0.22, *p* < 0.001), with the regression model being able to account for 16% of the variance in FM score (*F*(_2, 174)_ = 16.716, *p* < 0.001, see Table 3).

### 3.5. Mediation Analysis

Results of multiple regression analyses further justified the construction of two mediation models for testing the possible mediation effect of the OK score on the relationship between the frequencies of use of social and traditional media and TM and FM scores. Indeed, OK is the only predictor common to both TM and FM scores. 

**Figure 1:**
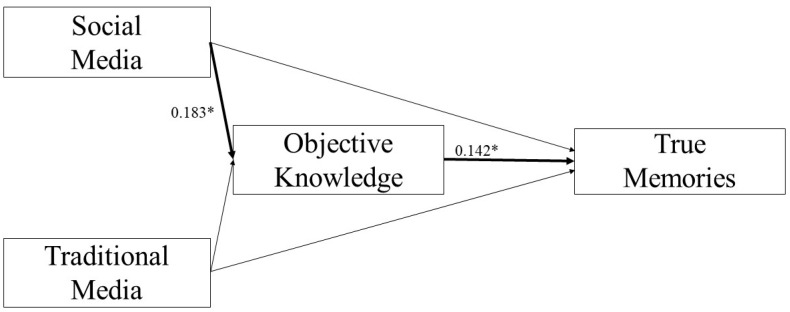
Scheme of the mediation effect of objective knowledge in the relationship between social media and true memories (* *p* < 0.05).

**Figure 2:**
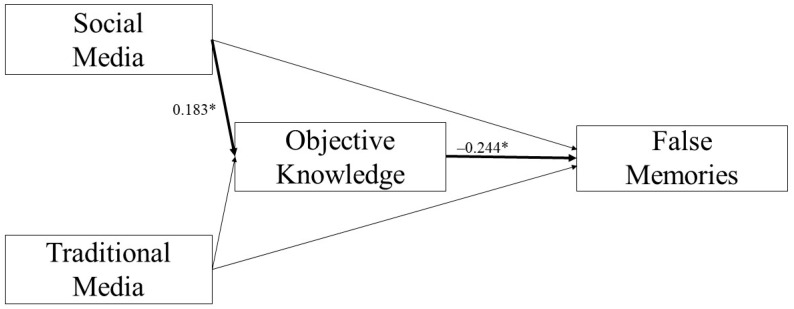
Scheme of the mediation effect of objective knowledge in the relationship between social media and false memories (* *p* < 0.05).

More use of social media was related to a higher OK score (*B* = 0.183, *p* = 0.025), but no significant relationship between the use of traditional media and OK score (*B* = 0.097, *p* = 0.299) was found. Furthermore, a higher OK score was associated with an increase in TM score (i.e., more true memories; *B* = 0.142, *p* = 0.002, Figure 1) and a decrease in FM score (i.e., less false memories; *B* = −0.244, *p* < 0.001, Figure 2).

The 95% bias-corrected CI based on 5000 bootstrap samples revealed that the indirect effects of the use of social media on both TM and FM scores through the OK score were significant (TM: Estimate effect: 0.026; 95% CI: 0.002, 0.057; FM: Estimate effect: −0.045; 95% CI: −0.092, −0.003).

## 4. Discussion

This study aimed at identifying the variables that most contributed to FMs for COVID-19-related fake news. Evidence provided by previous research was used to interpret our findings, although the main term of comparison for the discussion of our results was the study by Greene and Murphy [7], which was—to the best of our knowledge—the only contribution on this issue.

In line with Greene and Murphy, our participants recalled, on average, more true than fake news (1.89/4 vs. 0.75/4). Nevertheless, they showed poor memory also for the true news (*Surfaces, Immunization, Monoclonal antibodies*), apart from the *Symptoms* news (see Appendix A), which was correctly recalled by about 85% of the participants. This finding is likely due to the effectiveness of the awareness-raising campaign on the virus, which made information about the onset, the course, and the outcome of COVID-19 highly salient. 

Compared with previous investigations [7,26,27], the novelty of our study lies in having separately computed the use of traditional and social media to get information. In this respect, a higher use of traditional than social media was reported. Interestingly, traditional media were judged as the main source of information for most of the true news, whereas no significant mismatch was found between traditional and social media in participants who recalled the fake news. 

About the effect of individual variables, our study provides evidence not fully in agreement with Greene and Murphy’s study. Consistent with their findings, we found that fear of COVID-19 increased the TM score; however, we also found a negative association between fear that loved ones could contract COVID-19 and the number of FMs. These results further support the hypothesis that negative emotional valence is a powerful catalyzer of memory abilities, i.e., it can improve encoding and maintenance of information, thereby reducing reconstructive memory errors and thus the probability of generating FMs [7,12,14,52]. The selective effect that fear of loved ones contracting COVID had in reducing FMs might be explained by the empathy-mediated willingness to help [53]. In support of this hypothesis, converging evidence coming from neuroimaging studies suggesting that the medial prefrontal cortex (mPFC) is strictly involved in state empathy and prosocial behaviors [53,54,55] as well as in encoding and consolidation of memory traces [56], with particular reference to fear memories [56,57,58,59]. This neural overlap underlies a cognitive interaction across social and memory abilities. The mPFC was found to support the ability to recall the most appropriate motor and/or emotional response to specific events in a particular place and time. The ventral portion of mPFC is strongly interconnected with the anterior insula, which is involved in pain perception [60] and with the later habenula that, in concert with the Ventral Tegmental Area (VTA) and other regions of the dopamine network, plays a pivotal role in activating learned and adaptive responses to stressful/painful and to rewarding/motivationally salient events [61,62,63,64,65]. 

It is important to underline that the selective effect of the fear of loved ones contracting COVID-19—rather than contracting it personally—might be additionally explained by the fact that our sample consisted of young adults; therefore, they were less concerned about themselves than their older loved ones, who were more at risk for severe COVID-19 illness [28]. In sum, we hypothesize that our finding is likely explained by a prosociality-induced positive bias: the fear that a loved one may become ill, suffer, or even die leads to more carefully selecting the information disseminated about the disease, to filter them more thoroughly, and to construct more robust and durable mnestic traces in order to functionally cope with a potential emergency situation. When the goal is protecting others, people may be more motivated and committed, thus producing fewer cognitive errors. 

Previous research highlighted the role of cognitive ability in affecting FM formation, with a particular focus on analytic reasoning [21]. More specifically, it has been suggested that as cognitive skills increase, the probability of reporting FMs decreases [16]. Intriguingly, two studies conducted by Greene and Murphy’s research group, one on FMs for fake news among Brexit voters [27] and the other on FMs for COVID-19 fake news [7], showed that higher levels of analytical reasoning were associated with fewer FMs as well as fewer TMs. In other words, their participants were less likely to report a memory for any given news. Authors hypothesized that individuals with higher analytical skills may show a greater degree of suspicion towards all news. Indeed, they may require further evidence before reporting a memory for a piece of information that sounded familiar; this translates into a clear response bias [7,27]. Therefore, it has been suggested that higher analytic skills do not allow to efficiently discriminate between memories for true and fake news [27]. In addition, note that some studies reported no effect of cognitive abilities on FMs. For instance, in the study by Powers, Andriks, and Loftus [22], no relationship between susceptibility to FMs and cognitive/intellectual abilities, assessed through the Washington Pre-College Test (WPCT), was found. Still, Nichols and Loftus [24] found no correlation between FMs and the three-item version of the Cognitive Reflection Task (CRT), which was used to assess inhibition, analytical, and problem-solving capabilities. Interestingly, a seven-item version of the same task was used by Greene and Murphy in their study on COVID-19. Some methodological discrepancies (e.g., sample size, age of participants, assessment methods used) may explain the different patterns of results. Similar to Nichols and Loftus, we found no relationship between analytic skills and FM counts in a sample characterized by overlapping demographic features. Instead, compared with Greene and Murphy’s study, our participants were younger and only 18% of them demonstrated poor analytic skills (CET cut-off > 18, [46]). Conversely, participants tested by Greene and Murphy showed, on average, lower analytic abilities (*M* CRT score = 3.52, *SD* = 1.91, max CRT score = 7, [7]). Thus, dissimilar samples’ characteristics and assessment techniques may account for the difference in results. Future research should further explore the possible relationship between cognitive functioning and the construction of FMs, possibly using a standardized and ubiquitously accepted evaluation protocol. 

Neither the contribution of anxiety and depressive symptoms nor the role of personality traits in FM formation were explored in Greene and Murphy’s study. Previous research investigating the relationship between anxiety and FMs provided mixed results. Some studies found a positive association between anxiety and FMs [18,66], whereas other studies found no relationship [67,68,69]. Our results provide no evidence for a significant association between anxiety and memory biases and add to the previous literature suggesting that individuals suffering from anxiety (e.g., social anxiety, specific phobias) did not show a higher rate of FMs [69]. 

Studies assessing the effect of depression on FMs formation also provided conflicting results. Some evidence suggested that individuals with depression generated fewer FMs [15]; other investigations showed instead a positive relationship between depression and FMs [17,52,70]. Unlike previous research, we found no association between depression and frequency of FMs. This result might be due to the small number of participants (*n* = 10) obtaining a BDI score higher than normative cutoff (BDI ≥ 27, [40]). 

As for the role of personality traits, some studies reported a significant association between active [16] or negative coping strategies [16,18,19,20] and FM formation, while other ones detected no relationship between personality factors and FMs [24,71]. Our results add to this latter evidence. However, it is important to underline that a shorter inventory, as the COPE-NVI-25, might suffer from poorer reliability than longer assessment tools due to the lower scores’ variance. Further studies are needed to test this hypothesis. 

In the current study, the effect of both PK and OK on COVID-19-related information was also assessed. We found that OK was significantly associated with both TM and FM scores, whereas no effect of PK was detected. This result is at odds with previous evidence showing that PK might increase TMs [7] or memory biases [5] in subjects who rated themselves as experts. However, our finding reinforces the assumption that perceiving oneself as an expert on a certain topic does not necessarily mean that one actually is. Indeed, PK is closely related to the frequency of exposure to, and thus familiarity with, the information, whereas OK implies greater expertise, i.e., high-level competence and skills acquired on a particular task or within a given domain [72]. In line with this claim, several studies in the field of health care [73,74,75] and marketing [72,76] have reported weak or no association between PK and OK. Although, according to our results, FMs are unaffected by PK, overclaiming and overconfidence remain phenomena to be kept under control, especially when they involve public health issues. To achieve this goal, it would be necessary to promote the “knowledge calibration”, i.e., the correspondence between self-assessed and actual knowledge [72]. It is important to note that the different methodological approaches employed to evaluate PK and OK (i.e., one item rated on a Likert scale vs. an eight-item true/false questionnaire, respectively) could account for the observed dissociation. As a result, additional studies are needed to clarity the relation between PK and memory biases. 

Similar to Greene and Murphy, OK was found to be one of the best predictors of TM score and the only variable explaining a sufficient proportion of the FM score’s variance. In particular, OK increased the TM and decreased the FM score. Accordingly, a quantitative estimate of the knowledge about a certain topic is likely the most suitable measure of the ability to discriminate between true and fake news. Furthermore, this observation provides additional support to the MMF, which underlines the key role of expertise for critically and systematically evaluating the contents of an alleged memory [10]. 

Apart from OK, we found that both the frequency of use of traditional and social media predicted a significant increase in TM score. Furthermore, mediation analyses showed that OK mediated significantly the relationships between the use of social media and development of both TMs and FMs. Particularly, OK mediated positively the relationship between the use of social media and TMs, whereas the effect of OK was negative in mediating the relationship between the use of social media and FMs.

These findings suggest reconsidering the idea of social media as the main source of fake news, especially compared to traditional media that are usually considered more credible and objective [77]. Indeed, an increased use of social media was associated with a higher probability of reporting TMs as well as with a higher OK score. In turn, OK seems to play a crucial mediating role since it strengthens the formation of TMs and reduces the development of FMs following use of social media. Hence, the knowledge about the topic rather than the type of sources from which people choose to inform themselves would make the difference in the process of memories formation. This hypothesis is further supported by the lack of substantial differences between the use of traditional and social media among participants reporting FMs. The absence of an OK-driven mediation effect binding the relationships between traditional media and memory scores might be precisely justified by the tendency to consider information spread by traditional media as more realistic and truthful [77], thus bringing about a more passive approach without active recourse to one’s own knowledge.

The current study is not free from limitations. First, the sample size is fairly small, although the number of participants included in the study was far greater than the number estimated by the power analysis. Second, our sample included only university students. Thus, our results are biased by a limited external validity, even more so considering that differences in years of age and schooling can significantly affect the use of traditional and social media. Furthermore, our participants reported how often they checked social media, such as Facebook, Twitter, or Instagram, to inform themselves about the COVID-19 issue. However, by using social networks, it is possible to access either gossip pages, the “voice” of influencers/bloggers and public opinion, or the official pages of journalists, politicians, and experts in medical science. Still, it is possible to be redirected to official websites of newspapers/TV news or to the vision of partial/integral texts of scientific indexed papers recognized by the scientific community. Consequently, the mere estimation of the frequency of use of social media may not have been sufficient or still biased by the heterogeneity of accessible content. A further limitation might be traced to the fact that participants were not asked to specify how many people they knew that contracted COVID-19, which would have strengthened the role of the fear of the disease. Finally, although the fake news were constructed after a careful examination of news spread by media, it is difficult to rule out the possibility that participants had already generated FMs for similar episodes/news, so that our experiment would have uncovered pre-existing FMs rather than implanting them. However, we consider this scenario quite unlikely. 

## 5. Conclusions

The main findings of this study, which has examined the relationship between individual variables and FMs for COVID-19-related fake news, can be summarized as follows. Depression, anxiety, and personality traits seem to not affect FM formation. Furthermore, FMs appear also unaffected by reasoning skills, although the sample’s characteristics (i.e., highly educated young adults) may have produced a collapse of the variance. Overall, these findings add to previous evidence from mixed and confounding literature. 

The fear of loved ones contracting COVID-19, rather than the fear of contracting it personally reduced the FM count. This finding has been interpreted as the consequence of an empathy-mediated prosociality bias boosting memory ability. Furthermore, this is in line with the hypothesis that people experiencing negative emotions are less vulnerable to misleading information and less likely to form FMs. However, the young age of our participants should be taken into account, since it is to be expected that younger individuals are more fearful of their older loved ones contracting the virus than they are of themselves.

OK was found to be a significant mediator of the relationship between social media usage and FM construction. Individuals with greater knowledge about COVID-19 might be more able to discriminate between reliable and fictional information spread by social media; consequently, they might be less susceptible to memory biases. OK mediated also the relationship between social media and TMs, with higher OK being associated with more TMs. However, a higher frequency of use of social media explained, per se, the increase in TM score, with OK strengthening this relationship. In sum, our results suggest that social media should be cleared of the negative connotation that they are the main sources of disinformation; indeed, a lot depends on the knowledge of those who make use of social media. In this respect, political and health authorities, together with journalistic institutions, are encouraged to actively use their social channels but paying attention to disseminating clear, precise, and unambiguous information regarding the progress of the COVID-19 pandemic, especially since social media posts can reach people with limited medical knowledge, poor education, susceptibility to stereotypes or prejudices, or who favor heuristic reasoning schema.

## Figures and Tables

**Table 1:** Descriptive statistics.

Variables	Mean	SD	Range
TM score	1.89	0.97	0–4
FM score	0.75	0.84	0–3
Use of traditional media	3.56	1.19	0–5
Use of social media	3.18	1.37	0–5
PK score	3.19	0.86	1–5
OK score	5.20	1.45	1–8
Fear of contracting COVID-19	3.23	1.07	1–5
Fear of others contracting COVID-19	4.42	0.77	1–5
BDI score	12.15	7.69	0–37
BAI score	15.81	11.65	0–44
CET score	20.78	8.99	3–62
COPE—Avoidance Strategies	9.48	3.48	4–21
COPE—Transcendent Orientation	5.68	3.35	4–19
COPE—Positive Attitude	24.73	5.11	11–36
COPE—Social Support	18.95	5.87	5–30
COPE—Problem Solving	21.05	4.67	7–30

TM: True Memory; FM: False Memory; PK: Perceived Knowledge; OK: Objective Knowledge; BDI: Beck Depression Inventory; BAI: Beck Anxiety Inventory; CET: Cognitive Estimation Task; COPE: COPE-New Italian Version-25.

**Table 2:** Response rates for true and fake news and source types for the recalled news.

Responses Frequency (%)	Source Memory Frequency (%)	News			
		True news			
		Surfaces	Immunization	Monoclonal antibodies	Symptoms
I do not remember this		95 (53.7)	146 (82.5)	104 (58.8)	28 (15.8)
I remember this		82 (46.3)	31 (17.5)	73 (41.2)	149 (84.2)
	Traditional media	47 (57.3)	23 (74.2)	57 (78.08)	104 (69.8)
	Social media	35 (42.7)	8 (25.8)	16 (21.9)	45 (30.2)
		Fake news			
		Flu vaccine	Serological tests	Masks	Pets
I do not remember this		136 (76.8)	143 (80.8)	166 (93.8)	131 (74.0)
I remember this		41 (23.2)	34 (19.2)	11 (6.2)	46 (26.0)
	Traditional media	28 (69.3)	20 (58.8)	8 (72.7)	21 (45.6)
	Social media	13 (31.7)	14 (41.2)	3 (27.3)	25 (54.3)

**Table 3:** Results of simple and multiple regression analyses.

Predictors	Simple Regression Analysis				Multiple Regression Analysis			
	*B*	*t*	*p*	*R^2^*	*B*	*t*	*p*	*R^2^*
*True Memories*								
Use of traditional media	0.302	5.230	**<0.001**	0.135	0.243	4.253	**<0.001**	0.244
Use of social media	0.204	3.964	**<0.001**	0.082	0.131	2.646	**0.009**	
PK score	0.126	1.490	0.138	0.013				
OK score	0.188	3.868	**<0.001**	0.079	0.135	2.940	**0.004**	
Fear of contracting COVID-19	0.159	2.357	**0.020**	0.031	0.146	1.937	0.064	
Fear of others contracting COVID-19	0.204	2.149	**0.030**	0.026	–0.017	–0.157	0.875	
BDI score	−0.007	−0.765	0.446	0.003				
BAI score	−0.004	−0.660	0.510	0.002				
CET score	−0.009	−1.129	0.261	0.007				
COPE—Avoidance Strategies	0.013	0.605	0.546	0.002				
COPE—Transcendent Orientation	−0.021	−0.949	0.344	0.005				
COPE—Positive Attitude	−0.018	−1.289	0.199	0.009				
COPE—Social Support	0.003	0.265	0.792	0.000				
COPE—Problem Solving	−0.014	−0.911	0.363	0.005				
*False Memories*								
Use of traditional media	0.070	1.325	0.187	0.010				0.161
Use of social media	0.030	0.649	0.517	0.002				
PK score	0.072	0.980	0.329	0.005				
OK score	−0.225	−5.580	**<0.001**	0.151	−0.217	−5.364	**<0.001**	
Fear of contracting COVID-19	−0.088	−1.499	0.136	0.013				
Fear of others contracting COVID-19	−0.164	−2.006	**0.046**	0.022	−0.111	−1.451	0.149	
BDI score	0.001	0.138	0.890	0.000				
BAI score	0.005	0.923	0.357	0.005				
CET score	0.008	1.114	0.267	0.007				
COPE—Avoidance Strategies	0.007	0.383	0.702	0.001				
COPE—Transcendent Orientation	−0.003	−0.174	0.862	0.000				
COPE—Positive Attitude	0.007	0.563	0.574	0.002				
COPE—Social Support	−0.014	−1.321	0.188	0.010				
COPE—Problem Solving	−0.006	−0.456	0.649	0.001				

PK: Perceived Knowledge; OK: Objective Knowledge; BDI: Beck Depression Inventory; BAI: Beck Anxiety Inventory; CET: Cognitive Estimation Task; COPE: COPE-New Italian Version-25.

**Figure A1:**
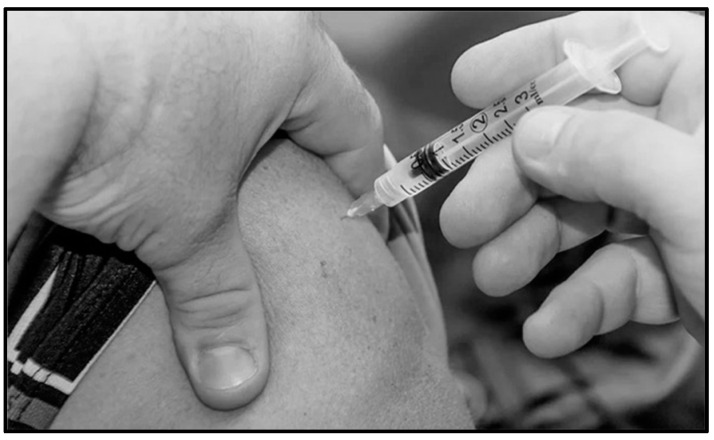
Flu vaccine.

**Figure A2:**
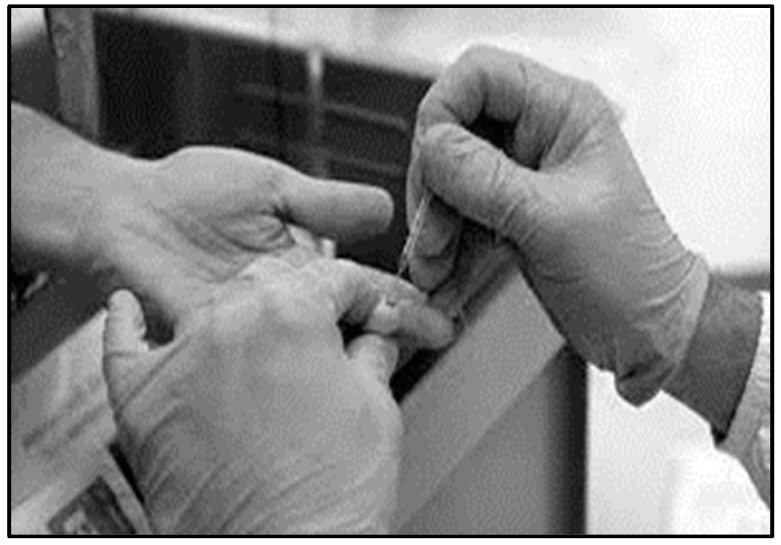
Serological tests.

**Figure A3:**
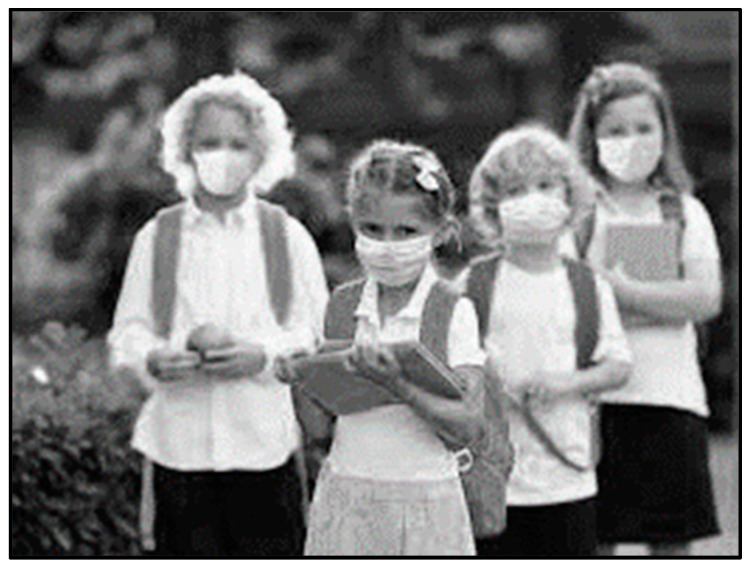
Masks.

**Figure A4:**
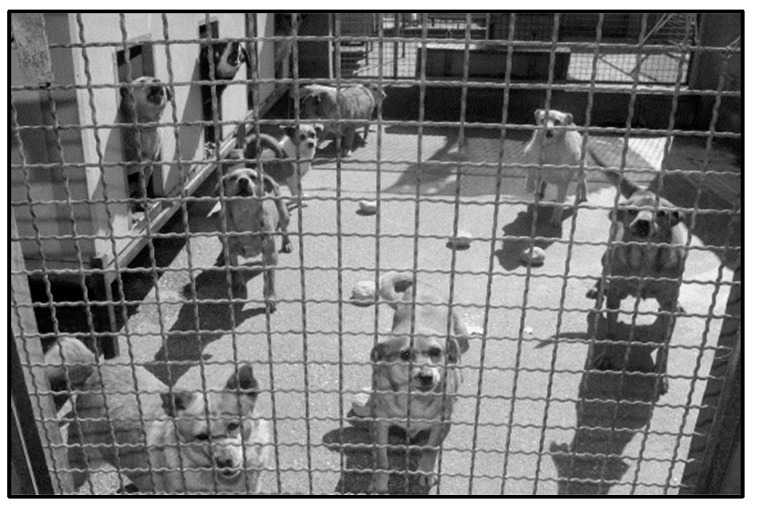
Pets.

**Figure A5:**
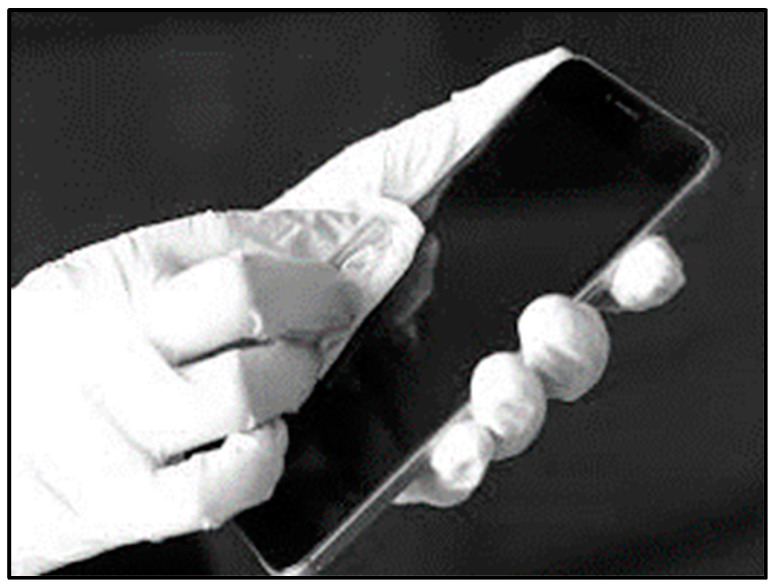
Surfaces.

**Figure A6:**
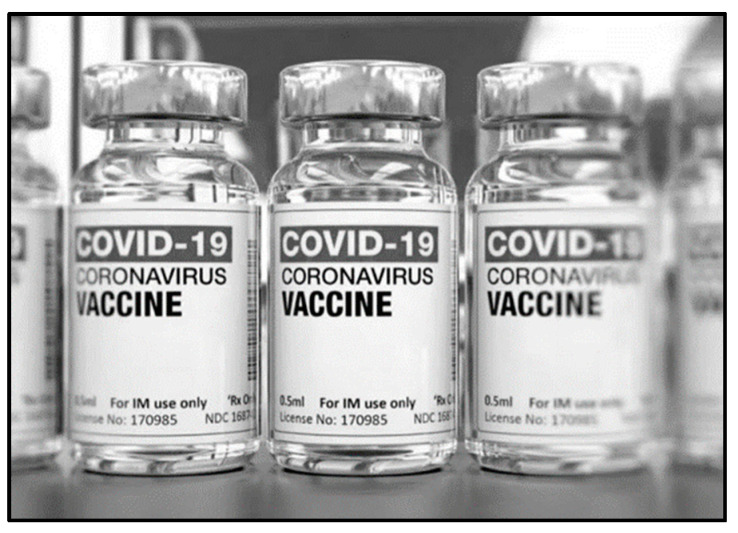
Immunization.

**Figure A7:**
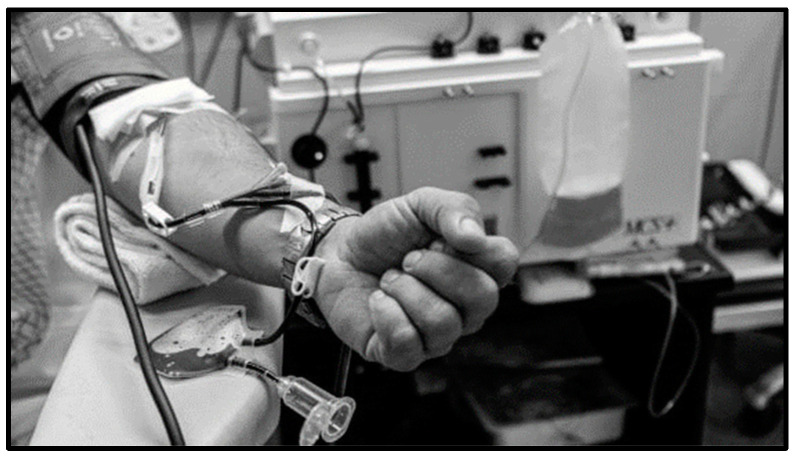
Monoclonal antibodies.

**Figure A8:**
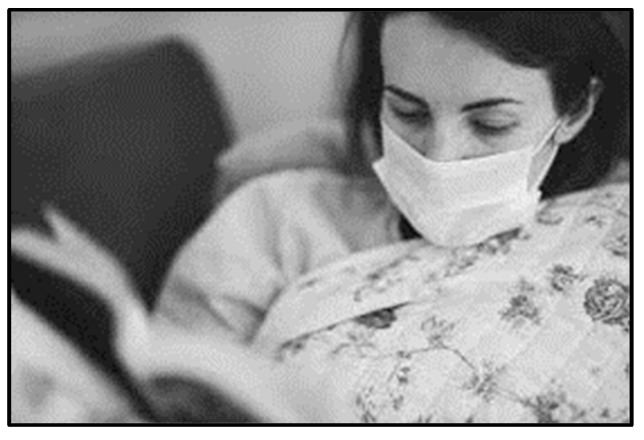
Symptoms.

## Data Availability

The data presented in this study are available on request from the corresponding authors.

## Appendix A

### Appendix A.1. Fake News

A recent study conducted by the Agostino Gemelli University Polyclinic in Rome has shown that 85% of patients who received flu vaccine were less likely to contract COVID-19 for at least 4 months following the vaccination.

In October 2020, the World Health Organization (WHO) announced that serological tests can, in the current state of the technological development, replace molecular and antigenic tests, as serological tests are able to detect both antibodies against the virus and the presence of COVID-19 in the body.

A study by the University College Dublin showed that 15% of Irish children under 10 years old experienced a temporary oxygen shortage and bacterial flora alteration following prolonged use of the masks.

In Belgium, some families decided to put down their domestic animals since they believed that the animals had gotten COVID-19 and could therefore transmit it to them.

### Appendix A.2. True News

According to a recent Australian study published in the Virology Journal, a sample of SARS-CoV-2, in conditions of ideal humidity and temperature, survives on some surfaces such as glass, steel and banknotes for up to 28 days.

Based on the data provided by the “Istituto Svizzero per gli Agenti Terapeutici”, the protection offered by vaccines administered so far in Italy is over 60% from seven days after the second dose.

In Italy, monoclonal antibodies are being experimented with, which are medications that have been used with discreet success in the United States to treat COVID-19 patients.

In some patients, the presence of residual symptoms after negativization has been found, such as fatigue, short breath, respiratory difficulties, and osteoarticular pain.
